# Heightened Levels of Maladaptive Daydreaming Are Associated With COVID-19 Lockdown, Pre-existing Psychiatric Diagnoses, and Intensified Psychological Dysfunctions: A Multi-country Study

**DOI:** 10.3389/fpsyt.2020.587455

**Published:** 2020-11-02

**Authors:** Eli Somer, Hisham M. Abu-Rayya, Adriano Schimmenti, Bariş Metin, Reut Brenner, Erika Ferrante, Buse Göçmen, Alessia Marino

**Affiliations:** ^1^School of Social Work, University of Haifa, Haifa, Israel; ^2^School of Psychology and Public Health, La Trobe University, Melbourne, VIC, Australia; ^3^Faculty of Human and Social Sciences, Kore University of Enna, Enna, Italy; ^4^Psychology Department, Üsküdar University, Istanbul, Turkey

**Keywords:** COVID-19, maladaptive daydreaming, fantasy, social distancing, self-isolation, quarantine

## Abstract

The COVID-19 pandemic has been spreading globally since December 2019, bringing with it anxieties, mortal risk, and agonizing psychological suffering. This study aimed to explore the relationship between maladaptive daydreaming (MD)—an addictive mental behavior to vivid fantasy associated with distress and functional impairment—and forced COVID-19 pandemic-related self-isolation and quarantine. Previous literature indicated that individuals employ MD for the regulation of distress and boredom, wish fulfillment, and entertainment experiences. The literature on the impact of the COVID-19 pandemic on mental health identifies a flareup in psychological difficulties in the general population. In this study we explored the associations between the pandemic threat and mental health indices among individuals with MD. We surveyed 1,565 adults from over 70 countries who responded to calls for participants posted in online MD communities and other general social media sites. Probable MD was determined based on an empirically derived cut-off score on a pertinent measure. After controlling for sociodemographic variables, a series of MANCOVAs, followed by *post-hoc* ANCOVAs, revealed that individuals with probable MD who were observing lockdown restrictions reported having spent more time in fantasy, experienced more intense and vivid daydreaming, and had a stronger urge to daydream than other participants. Similar statistical procedures indicated that, individuals with probable MD who reported pre-existing anxiety and depression disorders described a greater urge to daydream due to the pandemic and greater difficulty to control this addictive behavior. Compared to individuals with likely normal daydreaming, individuals with suspected MD reported more pandemic-attributed deterioration on a wide array of psychological distress indices. Our data show that the current worldwide pandemic threat is connected with an elevated intensity of this addictive form of mental activity, and that MD is associated with the exacerbation of psychological distress and dysfunction rather than with beneficial regulation of the experienced stressor.

## Introduction

### The COVID-19 Threat

The coronavirus disease 2019 (COVID-19) outbreak has evolved into a major global public health threat. According to the World Health Organization (WHO), as of 15 October 2020, 38,002,699 confirmed cases and 1,083,234 deaths have been reported in 216 countries and territories (1). The reported medical symptoms of COVID-19 are mostly respiratory and typically involve a dry cough, fever, fatigue, and loss of appetite, smell, and taste (2). Some patients develop acute respiratory distress syndrome that can become fatal in the most severe cases (3). COVID-19 has also been shown to affect other tissues, including the central nervous system (4–6). The coronavirus spreads mainly through respiratory droplets produced when an infected person coughs, sneezes, or talks. These droplets can land in the mouths or noses of people who are nearby, may be inhaled into the lungs or transferred to the facial cavities by touch with a contaminated hand. Spread is more likely when people are in close contact with one another, within about six feet or about two meters (7).

To break the chain of transmission of the coronavirus, the WHO has issued specific hygiene and social distancing guidelines (8) that have been implemented in most countries. In an attempt to minimize physical contact among people, many governments moved to close all places of socializing such as restaurants, bars, cafes, malls, theaters, and fitness centers, and even stopped public transportation. As the contagion rates peaked, authorities in numerous localities enforced a lockdown—a more stringent step involving a complete stoppage of any sort of public movement except essential services—a step that was escalated sometimes into a curfew, in anticipation of family gatherings during major holidays (9). Social distancing measures have included: (1) *self-quarantine*, a procedure imposed on individuals arriving from a country that has reported cases of coronavirus, or suspect they might have been infected by a COVID-19 positive person, and consequently have had to avoid human contact for 14 days while being observed for signs of the illness; and (2) *isolation* for those who have been tested positive or have developed symptoms. At the peak of the outbreak, when data for the study was collected, large portions of the population in most countries were forced to practice self-isolation under government lockdown orders.

### Psychological Consequences of COVID-19 on the General Population

The consequences of globally imposed precautionary measures of involuntary social distancing on mental health are complex. Community lockdown and broad compulsory isolation of citizens in the face of a mortal threat can generate a broad sense of existential uncertainty leading to a deterioration of health indices (10) impulsive shopping (11), and posttraumatic distress (12, 13). This can be intensified if extended family members need to be separated, and by uncertainties about disease spread and at-risk groups, an insufficient supply of elementary essentials, economic losses, ambiguous communications by the government (14–16), and rumors circulating in the social media (17).

Up to 38% of the general population affected by the pandemic restrictions seem to have experienced psychological distress (18). Individuals impacted by the COVID-19 threat may experience intense fear and anxiety (19) due to uncertainty about their state of health and develop obsessive-compulsive symptoms, such as repeated disinfecting, handwashing, and temperature checks (20), sleep disturbances (21), phobic anxiety and interpersonal sensitivity (22), and posttraumatic distress. Research indicates a positive correlation between the intensity of these outcomes and the duration of quarantine (23, 24).

The post-lockdown psychological effects include socioeconomic concerns and psychological symptoms associated with financial damages (14). Furthermore, stigmatization and societal rejection of quarantined and infected individuals have manifested in avoidance, suspicion, and discrimination (14). A recent meta-analysis indicated that the pooled prevalence of anxiety and depression in COVID-19 affected areas were 33% (95% confidence interval: 28–38%) and 28% (23–32%), respectively (25), illustrating the widespread morbid mental health consequences of the pandemic.

### Psychological Consequences of COVID-19 on Individuals With Pre-existing Mental Health Conditions

During periods of community threat, people with mental health disorders in general, and anxiety disorders in particular, are more vulnerable to a decline in their mental well-being (26). Individuals with pre-existing psychiatric disorders have a higher susceptibility to stress and therefore, could react more intensely to the COVID-19 threat, resulting in relapses or worsening of the pre-existing mental health problems (27). Indeed, persons with both preexisting physical and mental health conditions showed higher levels of anxiety (28) and depression following the pandemic declaration with the disease threat having a more distressing effect among patients with affective disorders (29–31). Gobbi et al. (32) demonstrated the significant worsening of psychiatric conditions as a result of the COVID-19 pandemic in a global sample of psychiatric patients. At least 50% of the assessed psychiatric patients showed elevated general psychological disturbance, risk for PTSD, and depression. During the peak of the COVID-19 pandemic, when strict lockdown measures were undertaken, psychiatric patients scored significantly higher than controls on measures of posttraumatic stress, depression, and anxiety (33). More than a quarter of these patients reported PTSD-like symptoms and moderate to severe insomnia. Furthermore, mental health patients were significantly more likely to report worries about their physical health, anger, impulsivity, and suicidal ideation (33).

The evidence concerning how people cope with the mental health outcome of the coronavirus threat is scarce. Beyond adherence to social distancing recommendations, respondents in one study distracted themselves by surfing the internet, listening to music, being active in meditation and prayer, and seeking social support (34). Acceptance and self-distraction were the most frequent coping strategies among participants with disabilities and chronic conditions (35).

Substance and behavioral addictions are often utilized by individuals to regulate their emotional distress. According to Khantzian's self-medication hypothesis (36), substance addiction functions as a compensatory means to regulate emotional pain, dysphoria, anxiety, and stress, a hypothesis that was supported by empirical evidence (37). Similar support to the self-medication hypothesis has also been found for behavioral addictions, such as gambling (38) and gaming disorder (39). What is the impact of the COVID-19 threat on addictive disorders?

### Psychological Consequences of COVID-19 on Individuals With Addictive Disorders

In recent commentaries scholars have expressed concerns that the current pandemic could increase the extent and severity of some addictive disorders (40–42). They suggested that the stressful measures imposed on the population to control the spread of the coronavirus could exacerbate some risk factors for the initiation, maintenance, and relapse of addictive behaviors. They also argued that the stress associated with social distancing is liable to increase the risk of resorting to substance use and behavioral addictions. Similar concerns were raised by Henry et al. (43) and McCann Pineo and Schwartz (44) who warned that the interaction of the COVID-19 pandemic with the U.S. opioid epidemic could have an overwhelming psychological impact on persons with substance use disorders.

Since broad disasters were prospectively associated with accelerations of alcohol use (45, 46), it is plausible that the current pandemic would also be linked with elevated substance and non-substance abuse (47). Accumulating evidence shows that COVID-19 distress was associated with elevated alcohol (48, 49) and other substance use (50). Experts [e.g., (27, 51)] have argued that the concept of addiction should not be restricted to the ingestion of substances. The literature suggests that behavioral addictions share several common features with substance addictions, such as natural history, phenomenology, or tolerance, supporting the inclusion of Addiction and Related Disorders in the *Diagnostic and Statistical Manual of Mental Disorders – fifth edition* [DSM-5; (52)]. This category includes non-substance-related disorders and pathological gambling. Moreover, internet gaming disorder was included in Section III of the DSM-5 as a condition for further study. Internet gaming disorder was said to originate as a coping mechanism against stress (53), and stress reduction was identified as an important motivational driver of excessive online gaming (54, 55). In a recent study, about half of the studied student sample reported that their gaming behavior had increased during the COVID-19 lockdown period and that gaming behavior was associated with examination-related stress (56). COVID-19 distress was also associated with elevated smartphone use (57), problematic gambling (58, 59) and Internet use (60).

In the current research, we wished to examine the relationship between COVID-19-related lockdown measures and a compulsive, potentially addictive, form of fantasy immersion called maladaptive daydreaming.

### Maladaptive Daydreaming

Despite its rewarding properties, the emerging disorder of maladaptive daydreaming (MD) can create dependency and distress and impairs important areas of functioning (61, 62). MD can serve numerous purposes, including active self-entertainment, a distraction from boredom, fantastical wish fulfillment, and self-soothing of emotional pain (63). Previous research demonstrated that persons recovering from substance use disorder (SUD) are more prone to engage in MD, thereby suggesting that both MD and SUD may share etiological and phenomenological characteristics (64).

This form of compulsive fantasy also shares similarities with some non-substance addictive behaviors, such as internet gaming disorder. Both could be considered escapist rewarding behaviors that cause intense craving for extension and repetition (62, 65). In fact, from a biobehavioral perspective, MD is characterized by a domain-specific compulsivity that has been recently posited as the principal psychopathological feature of addictive disorders (66). People with MD (maladaptive daydreamers, MDers) can absorb themselves daily in highly vivid daydreaming episodes lasting many hours. MD is characterized by an intense sense of presence that can generate powerful emotions. To maintain their daydreaming, many MDers feel a need to employ stereotypical movements [e.g., rocking or pacing; (61)]. Because MD sometimes involves vocalizations, gesturing, and kinesthesia, many need to protect their privacy when engaging in this mental activity. Social interactions require focusing attention resources on the outside. Socializing is, therefore, incompatible with MD, which drives some individuals to seek solitude for their daydreaming behavior. COVID-19 social isolation requirements may therefore intensify MD in two possible ways. First, in line with the function of other addictions, MD may also arguably be utilized for distress regulation (63, 67). Hence, according to the self-medication hypothesis (36), MD is likely to intensify during lockdown, especially as social isolation provides MDers with greater opportunities for the privacy needed to engage in this mental activity. Second, MDers who practice social isolation are likely to have fewer social obstacles to aid them in the control of MD, consequently weakening control over their mental habit. In line with these speculations, we hypothesized that:

H1: During COVID-19, self-isolation and self-quarantine would be positively associated with increased MD indices among MDers: time spent daydreaming (DD), the intensity of DD, the vividness of DD, and urge to DD; and would impede pre-COVID-19 efforts to restrain MD.

Previous data demonstrated that MD has a high likelihood of comorbidity with several mental disorders (68). Research has shown that concurrent mental problems can increase the likelihood of any addictive behavior to arise or intensify [e.g., (69)] and that addictive disorders and mood disorders often co-occur and display a negative reciprocal process (70). The indication that MD is not a mere intense variation of normal daydreaming comes also from its association with other mental disorders. We, therefore, hypothesized that:

H2: Compared to probable MDers without pre-pandemic mental health diagnoses, probable MDers with reported diagnoses would report a greater pandemic-related urge to daydream, more time spent in DD mode, higher intensity of DD, intensified vividness of DD, and a pandemic-related impedance of their pre-COVID-19 efforts to restrain MD.

Research has linked MD with a worsening of psychosocial distress amongst MDers [e.g., (62, 71)]. If MD were a helpful coping mechanism during the current pandemic, we would expect it to be associated with improved psychological wellbeing. However, if MD is a detrimental addictive behavior, we expect it to be ineffective as a means of coping and to co-occur with a myriad of unfavorable psychological and behavioral outcomes. Assuming that MD functions like an addictive disorder, we hypothesized that:

H3: Compared to non-MDers, probable MDers would report worsened pandemic-related deterioration on a wide array of psychosocial parameters: the ability to concentrate, life satisfaction, worries about the future, obsessions, compulsive habits, social anxiety, loneliness, depression, boredom, mental exhaustion, anger, emptiness, happiness, self-worth, and ability to maintain household chores.

## Materials and Methods

### Participants

Participants were recruited online. Invitations to take part in this study were posted on six English language Facebook communities, on one Italian and one Turkish Facebook community, all dedicated to MD peer support. Each MD Facebook community had between 400 and 2,000 members. Additionally, we sent email invitations to about 2,000 members of an English language MD email list. We also posted our invitation on a Reddit MD community hosting over 44,000 members. In an effort to recruit non-MDers, the authors circulated the call for participants in their own social media and email lists, asking readers to reshare the invitation. No reward was offered to participants. The sample for this study was comprised of 1,565 adults. Eight hundred seventy-two respondents (55.7%) met an evidence-based criterion for probable MD [i.e., MD = M ≥50 on the 16-item Maladaptive Daydreaming Scale, (68, 72)], and the rest (*n* = 693) were identified as non-MDers. MD scores of respondents with probable MD ranged between 50 and 100 (*M* = 70.74, *SD* = 11.02) and of those without probable MD ranged between 0 and 49.75 (*M* = 24.22, *SD* = 14.16). Respondents were residents of over 70 different countries (e.g., Australia, Canada, Egypt, France, Germany, Italy, Israel, Jordan, Japan, Kenya, Lebanon, Malaysia, Mexico, Morocco, The Netherlands, Norway, Philippines, Qatar, Romania, Russia, Saudi Arabia, Singapore, Spain, Sweden, Turkey, USA, UK). The countries most prevalent in our sample were the USA (22.9%), Italy (22.7%), Turkey (15.1%), UK (6.3%), and Canada (3.4%). Respondents' ages ranged between 18 and 80 years, with MDers being relatively younger than non-MDer respondents (*M* = 25.52, *SD* = 8.35; *M* = 36.37, *SD* = 15.11; respectively; *t*_(1562)_ = 18.01, *p* < 0.001). The MD and non-MD samples were predominantly female (77.9 and 75.5%, respectively). While no epidemiological data exist with regard to the sex ratio in MD, the disproportionate representation of women in our sample was in line with previous studies in the field (e.g., 65). Both groups were fairly well educated, with a tendency for probable MDers to have relatively less education compared to non-MDers ($\chi_{\left(2\right)}^{2}$ = 172.56, *p* < 0.001). The majority of probable MDers (65.83%) have completed either a bachelor's/post-graduate's degree or were studying toward such a degree, compared to 85.71% of their controls.

### Study Procedure

This study was approved by the Human Research Ethics Committee at the University of Haifa. In March 2020, the first author contacted the administrators of a large online English language MD community requesting assistance in rapidly developing a survey on the psychological sequelae of the COVID-19 pandemic among MDers. The administrators recruited 10 members of the community to serve as a focus group (73). The first author subsequently sent the group a list of possible effects of the extended social distancing measures and threats associated with the coronavirus. The original items on the suggested measure were from the literature on population reactions to threat [e.g., (12)], were based on the researcher's familiarity with MD, and were adapted to the coronavirus pandemic situation. The focus group discussed the list online over several days and provided the researchers with feedback that included modifications and suggested item additions. The final measure was designed to provide data for several studies conducted by the authors and included a few additional psychological scales.

The call for participants was posted on various online English, Italian, and Turkish MD community groups and was propagated by the researchers and the respondents on their respective social media networks. Only participants who were 18 years or older were included in the study. Potential participants were invited to visit an online informed consent form. After providing electronic consent, respondents were directed to online electronic surveys that took them between 15 and 20 min to complete. The survey was available in either English, Italian, or Turkish. Translation from the English source to Italian and Turkish was conducted by three native Italian and two native Turkish members of the research team. Data were collected between mid-April and mid-May 2020, a period in which the stay-at-home orders were still enforced globally.

### Measures

Respondents completed online self-report questionnaires, which included the following measures.

#### Demographics

Information was sought on participants' age, gender, country of residence, and education (elementary/junior/high school, bachelor's degree/student, post-graduate degree/student).

#### Behaviors During the Pandemic

Respondents were asked two binary (Yes/No) questions seeking information on whether they were required to practice certain preventive measures related to the pandemic lockdown. The target practices were defined to the respondents as follows: (1) self-isolation is employed when one is sick with symptoms of COVID-19 and is told by a health care provider to separate oneself from others, including from the people in the household, to the greatest extent possible, to prevent the spread of the virus; and (2) self-quarantine is taken to prevent the spread of a contagious disease like COVID-19 by asking people who were exposed to infected others to stay at home, a hotel room or a provided accommodation, and not leave for the period required to quarantine. No visitors are allowed into the quarantined home except for people who usually live in the household.

Maladaptive daydreaming. We employed Somer et al.'s (68) 16-item Maladaptive Daydreaming Scale (MDS-16) to gauge respondents' maladaptive daydreaming. A sample item is: ‘Some people feel distressed or concerned about the amount of time they spend daydreaming. How distressed do you currently feel about the amount of time you spend daydreaming?’ Respondents selected their responses on an 11-point Likert scale ranging from 0 to 100%, to indicate their daydreaming level. The MDS-16 demonstrated excellent internal reliability in the present study, with Cronbach's α =0.95.

#### Mental Health Diagnoses

Respondents were asked to indicate one or more mental health diagnoses assigned to them previously by their mental health professional. The list included “attention-deficit/hyperactivity disorder,” “social anxiety disorder,” “other anxiety disorders,” “obsessive-compulsive disorder,” “major depression,” and an open-ended “other mental health diagnoses” option.

#### COVID-19-Related Change in Daydreaming (DD) Indices

On a scale ranging from minus 10 (*extremely less*) to plus 10 (*extremely more*), with zero indicating the situation before the outbreak of the COVID-19 pandemic, respondents were required to rate the effects the COVID-19 pandemic on the (1) amount of time spent in DD, (2) intensity of DD, (3) vividness of DD, and (4) urge to DD.

#### COVID-19-Related Change in DD Control

Respondents were asked a binary (Yes/No) question about whether they were attempting to control their DD before the start of the COVID-19 pandemic. If answered in the affirmative, they were invited to indicate the degree to which the pandemic had impeded their restraining efforts on an 11-point Likert scale ranging from 0 (*not at all*) to 10 (*very much*).

#### COVID-19-Related Change in Psychosocial Functioning

On a scale ranging from minus 10 (*extremely worse*) to plus 10 (*extremely better*), with zero indicating the situation before the outbreak of the COVID-19 pandemic, the respondents were required to mark their current psychological condition on the following dimensions: the ability to concentrate, life satisfaction, worries about the future, obsessions, compulsive habits, social anxiety, loneliness, depression, boredom, mental exhaustion, anger, emptiness, happiness, self-worth, and ability to maintain household chores.

## Results

### H1: Self-isolation, Self-quarantine, DD Indices, MD Control

Controlling for gender, age, and education, Multivariate Analysis of Covariance (MANCOVA), followed by *post-hoc* ANCOVAs, was used to test differences in DD indices between probable MDers who were required to self-isolate or quarantine during the COVID-19 pandemic and probable MDers who were not required to take such actions. As hypothesized, the analyses indicated that reported DD indices were higher among suspected MDers who were required to self-isolate [*F*_(4,863)_ = 3.27, Λ = 0.98, *p* = 0.01] or self-quarantine [*F*_(4,863)_ = 5.23, Λ = 0.97, *p* < 0.001]. As shown in Table 1, *post-hoc* ANCOVAs revealed that MDers who self-isolated or self-quarantined reported more time spent in DD, experienced intensified DD, and had a stronger vividness experience of and urge to DD.

**Table 1:** *Post-hoc* ANCOVA results of the relationships between self-isolation/quarantine and DD indices.

	**Yes (*****n*** **=** **326)**		**No (*****n*** **=** **546)**					
	**Mean**	**SD**	**Mean**	**SD**	***F*_**(1, 866)**_**	***p***	***95%CI***	***d***
**Self-isolation during the COVID-19 pandemic**								
Time spent in DD	5.04	4.36	4.31	4.28	4.53	0.03	0.05−1.21	0.17
Intensity of DD	4.24	4.46	3.40	4.07	7.02	0.008	0.20−1.35	0.20
Vividness of DD	3.62	4.32	2.59	4.06	11.23	0.001	0.40−1.54	0.25
Urge to DD	4.89	4.43	4.00	4.16	8.12	0.004	0.26−1.43	0.20
	**Yes (*****n*** **=** **419)**		**No (*****n*** **=** **453)**					
	**Mean**	**SD**	**Mean**	**SD**	***F***_**(1, 866)**_	***p***	***95%CI***	***d***
**Self–quarantine during the COVID−19 pandemic**								
Time spent in DD	5.14	4.22	4.07	4.36	7.47	0.006	0.22−1.35	0.25
Intensity of DD	4.32	4.07	3.15	4.32	12.11	0.001	0.44−1.56	0.28
Vividness of DD	3.60	4.20	2.40	4.10	14.64	<0.001	0.53−1.64	0.29
Urge to DD	5.02	4.26	3.70	4.21	16.84	<0.001	0.62−1.76	0.31

*ANCOVA controlled for age, gender, and education; DD, Daydreaming; p, p–value; CI, Confidence interval of the difference; d, Cohen's d*.

The sub-MDer sample who were attempting to control their DD before the COVID-19 pandemic started (*n* = 433), reported that the COVID-19 pandemic impeded their restraining efforts to some degree, *t*_(431)_ = 2.02, *p* = 0.044, *M* = 5.40, *SD* = 4.14 (using the scale midpoint = 5 as a reference in the analysis). However, ANCOVA did not reveal any difference between MDers who practiced self-isolation or self-quarantine and those who did not in their perceived impediment of restraining efforts. Therefore, the hypothesized relationship between self-isolation or quarantine and impediment of pre-pandemic MD restraining efforts was not supported.

### H2: Mental Health Comorbidities, DD Indices, and MD Control

Major depression was reported by 26.1% (*n* = 226) of our respondents, social anxiety disorder was reported by 23.1% (*n* = 200) of our respondents, 25.1% (*n* = 217) reported having other anxiety disorders, 16.3% (*n* = 141) of our MDer sample stated that they had attention-deficit/hyperactivity disorder, 10.3% (*n* = 89) had obsessive-compulsive disorder, and 21.8% (n = 189) reported other mental health diagnoses. The relationships between mental health comorbidities and DD indices were tested using a series of MANCOVA, where the comorbidity acted as the between-subjects factor while controlling for age, gender, and education. This was followed by *post-hoc* ANCOVAs.

The comorbidity of MD with social anxiety disorder or other anxiety disorders was associated with higher DD indices [*F*_(4,856)_ = 3.10, Λ = 0.99, *p* = 0.027; *F*_(4,856)_ = 2.72, Λ = 0.99, *p* = 0.029, respectively]. Likewise, the comorbidity of MD with major depression or other mental health diagnoses was associated with higher DD indices [*F*_(4,856)_ = 4.70, Λ = 0.98, *p* = 0.002; *F*_(4,856)_ = 5.45, Λ = 0.98, *p* < 0.001, respectively]. As shown in Table 2, *post-hoc* ANCOVAs revealed that higher levels of DD intensity were associated with social anxiety diagnosis and other mental health diagnoses; an increased urge to DD was associated with social anxiety diagnosis, other anxiety disorders, major depression, and other mental health disorders. All other associations were statistically non-significant. Thus, the hypothesized relationship between comorbidity and DD indices was partially confirmed.

**Table 2:** *Post–hoc* ANCOVA results of the relationships between comorbidities and DD indices.

	**Yes (*****n*** **=** **200)**		**No (*****n*** **=** **665)**					
	**Mean**	**SD**	**Mean**	**SD**	*F*_(1, 859)*_	***P***	***95%CI***	***d***
**Social anxiety disorder**								
Time spent in DD	4.83	4.13	4.49	4.38	0.48	0.49	−0.43−0.91	0.08
Intensity of DD	4.31	4.10	3.50	4.26	4.67	**0.03**	0.07−1.40	**0.19**
Vividness of DD	3.39	4.11	2.83	4.20	2.02	0.15	−0.18−1.14	0.14
Urge to DD	5.19	3.90	4.13	4.31	7.91	**0.005**	0.29−1.63	**0.26**
	**Yes (*****n*** **=** **217)**		**No (*****n*** **=** **648)**					
	**Mean**	**SD**	**Mean**	**SD**	***F***_(1, 859)*_	***p***	***95%CI***	***d***
**Other anxiety disorders**								
Time spent in DD	4.76	4.39	4.50	4.30	0.91	0.34	−0.34−0.97	0.06
Intensity of DD	3.62	4.30	3.71	4.22	0.034	0.86	−0.71−0.59	0.02
Vividness of DD	2.97	4.31	2.95	4.15	0.014	0.91	−0.61−0.68	0.004
Urge to DD	5.06	4.10	4.15	4.27	6.61	**0.01**	0.20−1.50	**0.22**
	**Yes (*****n*** **=** **226)**		**No (*****n*** **=** **639)**					
	**Mean**	**SD**	**Mean**	**SD**	***F***_(1, 859)*_	***p***	***95%CI***	***d***
**Major depression**								
Time spent in DD	4.65	4.51	4.54	4.26	0.53	0.47	−0.41−0.88	0.03
Intensity of DD	4.03	4.12	3.56	4.28	2.88	0.09	−0.09−1.19	0.11
Vividness of DD	3.08	4.28	2.91	4.15	0.47	0.50	−0.42−0.86	0.04
Urge to DD	5.24	3.99	4.07	4.29	12.67	** <0.001**	0.52−1.80	**0.28**
	**Yes (*****n*** **=** **189)**		**No (*****n*** **=** **676)**					
	**Mean**	**SD**	**Mean**	**SD**	***F***_(1, 859)*_	***p***	***95%CI***	***d***
**Other diagnoses**								
Time spent in DD	4.60	4.62	4.56	4.24	0.53	0.47	−0.43−0.94	<0.01
Intensity of DD	4.23	4.13	3.53	4.26	5.70	**0.017**	0.15−1.51	**0.17**
Vividness of DD	3.41	4.36	2.83	4.13	3.79	0.052	−0.01−1.35	0.14
Urge to DD	5.47	4.12	4.07	4.23	17.56	** <0.001**	0.77−2.13	**0.34**

ANCOVA controlled for age, gender, and education; DD, Daydreaming; p, p-value; CI, Confidence interval of the difference; d, Cohen's d; * there were 7 missing cases for each diagnosis. Bold values refer to statistically significant differences between the groups.

To test the relationships between mental health comorbidities and impediment of restraining MD efforts among the MDer sample, ANCOVAs were conducted on the sub-MDer sample who were trying to control their DD before the COVID-19 pandemic started (*n* = 433). Age, gender, and education were the covariates. Those who indicated a major depression diagnosis reported that their efforts (*M* = 6.40, *SD* = 3.73) were hampered due to the pandemic [*F*_(1,421)_ = 6.26, *p* = 0.01, Cohen's *d* = 0.31], more than the impediment levels (*M* = 5.20, *SD* = 4.09) reported by those who did not have major depression. Likewise, higher levels of impeded efforts (*M* = 6.22, *SD* = 3.06) were reported by those who had other anxiety disorders [*F*_(1,421)_ = 3.71, *p* = 0.049, Cohen's *d* = 0.25], compared to the reported impediment levels (*M* = 5.29, *SD* = 4.25) by those without other anxiety disorders. Thus, the hypothesized relationship between comorbidity and MD restraining efforts was also partially confirmed.

### H3: Comparisons Between MDers and non-MDers Across Psychosocial Dysfunctions

MANCOVA and a series of *post-hoc* ANCOVAs controlling for age, gender, and education were also employed here to test differences between the MDer and non-MDer groups. In support of the third hypothesis, psychosocial indicators emerged as different between the MDer and non-MDer groups, *F*_(15,1485)_ = 19.50, Λ = 0.88, *p* < 0.001. As shown in Table 3, MDers' self-reported ability to concentrate and life satisfaction has significantly deteriorated due to the pandemic; this was also true for MDers' worries about the future, obsessions, compulsive habits, social anxiety, loneliness, depression, boredom, mental exhaustion, anger, emptiness, and lower happiness, self-worth, and ability to maintain household chores.

**Table 3:** *Post-hoc* ANCOVA results of the MDers and non-MDers' differences in worsened psychosocial indicators due to the COVID-19 pandemic.

	**MDers (*****n*** **=** **838)**		**Non-MDers (*****n*** **=** **666)**					
	**Mean**	**SD**	**Mean**	**SD**	*F*_(1, 1499)^*^_	***p***	***95%CI***	***d***
Ability to concentrate	−3.41	4.35	−1.28	4.42	45.14	<0.001	−2.21— −1.21	0.49
Life satisfaction	−3.28	4.64	−1.28	4.66	22.36	<0.001	−1.80— −0.75	0.43
Future worries	−2.19	5.97	−0.12	5.05	53.08	<0.001	−2.99— −1.72	0.37
Obsessions	−1.64	4.45	0	3.18	54.47	<0.001	−2.15— −1.25	0.42
Compulsive habits	−1.43	4.09	−0.55	3.28	19.95	<0.001	−1.31— −0.45	0.24
Social anxiety	−0.88	5.12	−0.31	4.28	5.23	0.019	−1.20— −0.11	0.12
Loneliness	−1.98	5.60	−1.05	4.74	5.09	0.024	−1.29— −0.09	0.18
Depression	−2.18	4.76	−0.32	3.97	40.07	<0.001	−2.14— −1.13	0.42
Boredom	−1.75	5.66	−0.09	5.03	22.35	<0.001	−2.10— −0.87	0.31
Mental exhaustion	−2.44	5.79	−0.42	4.36	46.92	<0.001	−2.67— −1.48	0.39
Anger	−0.93	4.21	−0.26	3.83	7.99	0.005	−1.13— −0.20	0.17
Emptiness	−2.16	5.24	−0.27	4.27	36.19	<0.001	−2.25— −1.14	0.40
Happiness	−1.79	4.19	−0.40	3.85	24.39	<0.001	−1.63— −0.70	0.35
Self-worth	−2.36	4.69	0.27	4.10	69.13	<0.001	−2.66— −1.64	0.60
Household chores' Maintenance ability	−1.38	4.92	1.82	4.22	110.58	<0.001	−3.35— −2.30	0.70

ANCOVA controlled for age, gender, and education; MDer, Maladaptive Daydreamer; p, p-value; CI, Confidence interval of the difference; d, Cohen's d; each indicator was measured on a scale ranging from minus 10 to plus 10, with zero indicating the situation before the outbreak of the COVD-19 pandemic;

* *there were 1,509 valid list wise cases used by MANCOVA and post-hoc ANCOVAs, 34 and 27 list wise missing cases in the MDer and non-MDer groups, respectively; the distribution mode for each of the variables in the non-MDers group is zero*.

## Discussion

Our data lent support to the first hypothesis of this study and showed that daydreaming addiction indices were higher among participants with suspected MD who adhered to the self-quarantine orders and socially self-isolated. Under lockdown, probable MDers reported a greater urge to daydream, spent more time absorbed in daydreaming, and experienced more intensified and more vivid fantasies. Regardless of their practice of social distancing, those who attempted to control their MD before the outbreak of the pandemic reported that the COVID-19 lockdown impeded their efforts to regulate their habit.

Several factors could account for why MD activity increased during the social distancing enforced at the height of the pandemic. Many people in MD communities described how the restrictive disease-containment measures affected them. Common themes were worry, distress associated with the enforced home confinement and with forced intimacy, disruption to routines, inability to distract from their urges to daydream, and boredom, indicating that their preferred way of coping with the distressing situation was MD. These themes were clearly echoed in the e-mails spontaneously sent by MDers participating in the study to the research team during data collection. It is likely that our respondents behaved along the lines of Khantzian's self-medication hypothesis (36) and resorted to their non-substance addiction to relieve the coronavirus and lockdown distress. While hypothetically effective in the short run, this form of coping strategy is essentially avoidant. Avoidant coping style is associated with subsequent elevated anxiety and depression in animals (74) and humans alike (75), and can also affect a range of psychological functions among MDers, as we will demonstrate below. The documented increase in MD behavior is in line with recent data presented by Rodriguez et al. (49) who showed that psychological distress associated with the COVID-19 pandemic is consistently related to alcohol use indices. Our findings also correspond with the evidence presented by Elhai et al. (57) who showed that COVID-19 anxiety correlated positively with the severity of another escapist behavior: problematic smartphone use. In a similar vein, the lockdown, following the spread of COVID-19, was also associated with an increase in internet gaming behavior (56).

About one of every four participants in our study who was classified as a probable MD reported a prior diagnosis of major depression or an anxiety disorder. ADHD and OCD were reported by 16.5 and 10.7% respectively, and about one of every five MDers reported a prior diagnosis of another psychiatric disorder. These data support evidence from an earlier study that showed a high likelihood for MD to occur with other psychiatric disorders (68).

In support of our second hypothesis, we also found that probable MDers with reported major depression and those with reported anxiety disorders experienced an increase in MD indices and more difficulties in controlling their behavioral addiction due to the pandemic. From this perspective, MD with co-occurring mental health problems seem to behave like substance use disorders with comorbidities. For example, Najt et al. (76) reviewed the literature and reported poorer outcomes for substance use disorders with co-occurring mood disorders. Compared with the community sample, individuals with dual diagnoses reported more addictive behavior problems, engaged in more substance use to cope, experienced higher relapse rates, experienced more negative life (77), and subsequently incurred higher psychiatric treatment costs (78).

From a biological perspective, the link between MD and psychiatric comorbidities might be associated with an increased default mode network activation. Neuroimaging studies showed that daydreaming is associated with the brain's default mode network [DMN, (79)]. Connectivity and activity alterations in this neural network was also linked to several psychiatric disorders such as depression (80). Future research should investigate if MDers with a comorbidity of depression are more susceptible to engage their DMN during psychological stress.

During the pandemic lockdown and compared to the comparison group, probable MDers reported a significant deterioration in a wide array of psychological indices. Compared to the pre-lockdown period, probable MDers experienced more concentration difficulties, lower life satisfaction, more worries about the future, and more obsessions, compulsive habits, social anxiety, loneliness, depression, boredom, mental exhaustion, anger, emptiness, as well as lowered happiness, decreased self-worth, and impaired ability to maintain household chores. These findings provide further evidence that MD is a mental disorder with typical psychopathological hallmarks of distress and dysfunction. The unfavorable MD outcome resembles that of known escapist behavioral addictions. For example, internet gaming addiction, a fantasy-based escapist habit, was related to lowered academic performance, decreased self-confidence, and lowered self-esteem (81). Pathological internet use, in general, was linked with social withdrawal, self-neglect, and family problems (82), while gambling disorder was linked with stress-related medical conditions, lower work productivity, strained social relationships, guilt, shame, depression, anxiety and, substance use (83).

Some study caveats should be acknowledged. First, while our study recruited a large international sample, generalizability of the findings cannot be ascertained due to sampling limitations. Second, although our questions required respondents to assess psychosocial change in comparison to pre-pandemic times, thereby providing us with information about change, the cross-sectional design of the study limits our ability to infer causal relationships among the measured variables. Third, as measured by Cohen's *d*, the strengths of the relationships between the study variables were moderate to small. Lastly, the timely performance of clinician-administered diagnostic interviews to accurately identify MDers was prohibitive in our very large study sample. Therefore, our study is limited by resorting to an empirically derived benchmark to identify probable MDers.

## Conclusions

This study contributes to the understanding of a newly emerging construct of MD as a behavioral addiction to fantasy and absorption. We showed that MD displays similar characteristics and stress outcomes to those observed in other substance and non-substance addictions. We further demonstrated that although MD may have its origins as a normal, entertaining, and seemingly harmless form of coping, its intensified activation in the face of a major stressor resulted in a marked deterioration in a wide range of psychosocial functions. The ability to test this mental habit under real-time stress provided us with a rare opportunity to employ an objective stressor, rather than relying on the respondents' memory and its inherent biases. The current study also provides further evidence on the adverse mental health effects of the coronavirus pandemic, an unprecedented existential threat to humanity. One immediate implication derived from the current research is that mental health professionals should screen for MD to prevent a possible worsening of this addictive mental behavior and the flareup of resultant psychosocial impairments. Based on the accumulating data, and in line with the recommendation of Sani et al. (84), we advocate the immediate inclusion of mental health experts in policy task forces working on the prevention of a secondary mental health pandemic.

## Data Availability Statement

The raw data supporting the conclusions of this article will be made available by the authors upon request without undue reservation.

## Ethics Statement

The studies involving human participants were reviewed and approved by University of Haifa Faculty of Welfare and Health Studies Committee of Ethics in Human Research. The participants provided their written informed consent to participate in this study.

## Author Contributions

ES: conception and design of the work, data collection, data interpretation, drafting the article, critical revision of the article, and final approval of the version to be published. HA-R: conception of the work, data collection, data analysis and interpretation, drafting the article, critical revision of the article, and final approval of the version to be published. AS: conception of the work, data collection, data interpretation, and critical revision of the article. BM: conception of the work, data collection, data interpretation, and critical revision of the article. RB, EF, BG, and AM: conception of the work, data collection and entry. All authors contributed to the article and approved the submitted version.

## Conflict of Interest

The authors declare that the research was conducted in the absence of any commercial or financial relationships that could be construed as a potential conflict of interest.
